# Feeling Hot but Accepting It: Relationship Between Thermal Perception-Acceptance Discrepancy & Physical Symptoms

**DOI:** 10.1093/geroni/igaf122.792

**Published:** 2025-12-31

**Authors:** Molly Lin, Rachel Leung, Helene Fung

**Affiliations:** University of Zurich, Zürich, Switzerland; The Chinese University of Hong Kong, Hong Kong, China (People’s Republic); The Chinese University of Hong Kong, Hong Kong, China (People’s Republic)

## Abstract

The increasing severity of hot weather is considered an emerging threat to public health, especially for older adults. While research has demonstrated the negative effects of hot temperatures on older adults’ physical well-being, limited research has examined whether their perceptions of the temperature provide distinct predictions of physical well-being beyond the objective temperature, let alone the discrepancies between thermal perception and thermal acceptance. A time-sampling study was conducted during the hottest months (May – September) in 2021 and 2022 in Hong Kong. A total of 321 participants (k = 9,282) aged 60 and above reported their physical symptoms, thermal perception, and thermal acceptance three times a day across ten days and wore sensors that tracked the instantaneous temperature of their immediate environment. Multilevel models showed that thermal perception, thermal acceptance, and the perception-acceptance discrepancy distinctively predicted the likelihood of experiencing physical symptoms, after controlling for the objective temperature. A larger perception-acceptance discrepancy predicted a higher likelihood of reporting physical symptoms. Factors influencing this perception-acceptance discrepancy were explored. These findings provide implications for promoting heat adaptation behaviors among older adults.

